# Factors That Influence Length of Membership in a Customer-Driven Organization: A Village

**DOI:** 10.1093/geroni/igaa057.161

**Published:** 2020-12-16

**Authors:** Joonyoung Cho, Ruth Dunkle, Karen Harlow-Rosentraub

**Affiliations:** University of Michigan, Ann Arbor, Michigan, United States

## Abstract

Membership is a critical feature of the survival of customer-driven organizations. As a membership-driven organization based on neighbors helping neighbors, many Villages express difficulty in having enough members and lack confidence in sustainability. This is the first study examining the association between length of membership and motivation for becoming a Village member. ShareCare, the first Village, was founded in 1994. We used an open-ended questionnaire to gather information from a representative sample of current Sharecare members (N=100). Three researchers were involved in coding responses with discrepancies resolved via collaborative discussion. Length of membership was categorized as: less than 10-years, and more than a 10-year membership. Motivations to join membership in ShareCare were categorized as: instrumental, social, and altruistic. We conducted three separate logistic regressions with covariates controlled to examine associations between length of membership and various motivations to become a ShareCare member. While the most frequent reason for joining was instrumental where the member would receive service (e.g., care coordination, and home visit), the least motivation for joining was altruism, where the member could help other members (e.g., running errand, and lawn care). More than a 10-year membership was not associated with social or instrumental motivation to join ([OR] 0.50, p = 0.27, [OR] 0.94, p = 0.95) whereas more than a 10-year membership was associated with altruistic motivation to join ([OR] 5.31, p = 0.02). Our findings provide guidance regarding motivating members to join and maintain membership in a consumer-driven organization.

